# The contribution of alcohol consumption and smoking to educational inequalities in life expectancy among Swedish men and women during 1991–2008

**DOI:** 10.1007/s00038-017-1029-7

**Published:** 2017-08-23

**Authors:** Olof Östergren, Pekka Martikainen, Olle Lundberg

**Affiliations:** 1 Centre for Health Equity Studies, Stockholm University/Karolinska Institutet, Sveavägen 160, 106 91 Stockholm, Sweden; 2 Department of Social Research, University of Helsinki, Helsinki, Finland

**Keywords:** Mortality, Health inequalities, Alcohol, Smoking, Registers

## Abstract

**Objectives:**

To assess the level and changes in contribution of smoking and alcohol-related mortality to educational differences in life expectancy in Sweden.

**Methods:**

We used register data on the Swedish population at ages 30–74 during 1991–2008. Cause of death was used to identify alcohol-related deaths, while smoking-related mortality was estimated using lung cancer mortality to indirectly assess the impact of smoking on all-cause mortality.

**Results:**

Alcohol consumption and smoking contributed to educational differences in life expectancy. Alcohol-related mortality was higher among men and contributed substantially to inequalities among men and made a small (but increasing) contribution to inequalities among women. Smoking-related mortality decreased among men but increased among women, primarily among the low educated. At the end of the follow-up, smoking-related mortality were at similar levels among men and women. The widening gap in life expectancy among women could largely be attributed to smoking.

**Conclusions:**

Smoking and alcohol consumption contribute to educational differences in life expectancy among men and women. The majority of the widening in the educational gap in mortality among women can be attributed to alcohol and smoking-related mortality.

## Introduction

Social inequalities in health persist in modern welfare states. Perhaps surprisingly, the comprehensive welfare states in the Nordic countries do not exhibit smaller social inequalities in health compared to other European countries (Bambra 2011; Eikemo et al. 2008; Mackenbach et al. 2008, 2016). Both Bambra (2011) and Mackenbach (2012) suggest that health-related behavior may partly explain why Nordic welfare programs have not reduced social inequalities in health. The aim of this study is to assess the contribution of two key health behaviors—alcohol and smoking—to the time trends in social inequalities in life expectancy in Sweden.

Differences in health-related behavior are among the suggested explanations for the educational gradient in mortality (Cutler and Lleras-Muney 2010; Mirowsky and Ross 2003). Education may shape health-related behaviors because it is a strong determinant of both material and social circumstances (Mirowsky and Ross 2005; Ross and Wu 1995) and it helps individuals with information-seeking and decision-making (Mirowsky and Ross 2003). The low educated may be disproportionately exposed to difficult life circumstances and economic difficulties and unemployment have been linked to smoking (Backlund et al. 1996; Bloomfield et al. 2006) and drinking (Backlund et al. 1996; Wagstaff and Van Doorslaer 2000). Smoking and drinking have also been suggested to be part of coping strategies to deal with stress (Seeman et al. 1988; Slopen et al. 2013; Taylor and Sirois 2012). Previous studies suggest that individuals with high education are more likely to attempt to quit smoking and are also more likely to be successful in quitting (Reid et al. 2010). Drinking and smoking may also be part of processes separating and consolidating social groups (Seeman et al. 1988; Skeggs 1997; Slopen et al. 2013). Previous studies have demonstrated associations between education and smoking and harmful drinking (Cutler and Lleras-Muney 2010; Laaksonen et al. 2003) and educational gradients in smoking- and alcohol-related mortality have been reported for Sweden in international studies (Kulik et al. 2014; Mackenbach et al. 2008, 2015). Alcohol- and smoking-related mortality have been found to represent an important part of the level and trends in educational inequalities in mortality in Denmark (Koch et al. 2015) and Finland (Martikainen et al. 2013).

Relative educational inequalities in mortality have been increasing faster among women than among men in Sweden (Östergren 2015), in part due to comparatively small gains in life expectancy among low educated women (Statistics Sweden 2011). During recent decades, smoking shifted from being more common among men to being more common among women (Foulds et al. 2003) and lung cancer incidence have declined among men but increased among women (Devesa et al. 2005). International comparative studies have indicated that smoking accounts for a relatively small proportion of mortality in Sweden, particularly among men (Rodu and Cole 2004). Comparing educational inequalities in smoking-related causes of death, Kulik et al. (2014) found comparatively small inequalities among Swedish men, but not among women (Kulik et al. 2014). Giskes et al. (2005) observed decreasing levels of smoking among Swedish men at all educational levels and high educated women between 1985 and 2000, while smoking among low educated women increased during the period (Giskes et al. 2005).

A larger proportion of mortality among Swedish men than among women is attributable to alcohol (Foulds et al. 2003). Although major public health surveys have found small differences in heavy episodic drinking by education among Swedish men and women (Bloomfield et al. 2006; Backhans et al. 2015), a persisting educational gradient in mortality from alcohol-related causes among Swedish men and women were reported in a recent study by Mackenbach et al. (2015). The low educated may be more susceptible to harmful effects of alcohol consumption and smoking (Nordahl et al. 2014) since this group tend to have worse health in general (Mirowsky and Ross 2003) and is more likely to engage in multiple negative health behaviors (Laaksonen et al. 2003). It is possible that alcohol-related mortality may be higher among the lower educated even at similar levels and patterns of consumption. Another possibility is that survey data may not fully capture all aspects of alcohol consumption since it measures consumption at one point in time and may underestimate true levels and patterns of life-time consumption.

Based on longitudinal data covering the total population of Sweden with consistent measurement of education and mortality over time and no loss to follow-up, the aim of this study is to estimate the contribution of alcohol consumption and smoking to the level and time trends in educational inequalities in life expectancy among Swedish men and women during 1991–2008.

## Methods

### Data

The data consisted of an open cohort including all Swedish-born individuals between the ages 30 and 74 during the period 1991–2008, constructed from the total population register. Cause of death was collected from the cause of death register. The educational register was initiated in 1990 and is kept on individuals of 74 years of age and younger. The lower age limit was set to 30 when most individuals have completed their education and thus the age range of 30–74 was used throughout the study period. Data on education for Swedes born outside of Sweden are often missing and this group was, therefore, excluded. Since sociodemographic variables are compiled at the end of the year, no information on individuals’ education for the deceased in 1990 could be collected. The beginning of mortality follow-up was thus set to 1991-01-01 and finished in 2008-12-31 which was the last complete year of mortality data available for analysis.

Education was classified into three groups based on the ISCED-97 classification system. ISCED 0-2, lower secondary education or lower (“Low”), ICED 3–4, upper secondary education (“Intermediate”), and ISCED 5–6, tertiary education (“High”). Age was divided into 5-year age groups. To ensure stable mortality estimates, the data was collapsed into 3-year periods; 1991–1993, 1994–1996, 1997–1999, 2000–2002, 2003–2005, and 2006–2008 (Table 1).

**Table 1:** Number of person years and deaths included in the data, men and women, Sweden, 1991–2008

	Men		Women	
	Person years	Deaths	Person years	Deaths
1991–1993	6,113,668	52,740	6,135,829	31,656
1994–1996	6,198,869	47,556	6,190,005	29,211
1997–1999	6,301,691	43,616	6,252,594	26,719
2000–2002	6,400,246	40,146	6,317,563	26,097
2003–2005	6,524,992	38,867	6,410,945	25,243
2006–2008	6,620,855	37,645	6,482,298	24,633
Total	38,160,321	260,570	37,789,234	138,926

### Methods

The number of alcohol-related deaths was estimated by identifying deaths that included any alcohol-related cause as the underlying or the contributing cause of death. The inclusion of a cause of death on the death certificate, either as the underlying or a contributing cause, indicates that the cause was considered as part of the causal chain leading to death by the physician responsible for writing the death certificate. Furthermore, in ICD-10 there is a set of exceptions to the rules regulating when a cause should be reported as underlying and contributing, affecting about 20% of all deaths (The National Board of Health and Welfare 2010). Consequently, all deaths including at least one alcohol-related cause of death are classified as alcohol-related deaths in this study. Over the study years, the underlying cause of death was alcohol-related on average in 43% of all the alcohol-related deaths. ICD-9 was used in 1991–1996, and ICD-10 was used in 1997–2008. The following causes were classified as alcohol-related in ICD-9: alcohol-related psychosis and mental disorders (291A-F, 291W, 291X), alcohol dependence (303), alcohol abuse (305A), alcohol-related nerve damage (357F), alcoholic myopathy (425F), alcoholic gastritis (535D) and alcoholic liver disease including liver cirrhosis (571A–D). In ICD-10, the following causes were classified as alcohol-related: alcohol-related psychosis and mental disorders (F10.0–9), alcohol-related nerve damage (G31.2), alcohol-induced epilepsy (G40.5), alcoholic myopathy (G72.1), alcoholic gastritis (K29.2), and alcoholic liver disease including liver cirrhosis (K70.0–4, K70.9), alcoholic pancreatitis (ICD-10: K85.2, K86.0) and accidental alcohol poisoning (ICD-10: X45). The proportion of alcohol-related deaths in 1996 and 1997 was similar (5.1 and 5.3%, respectively), indicating that the shift between ICD-9 and ICD-10 did not substantially influence the likelihood of classifying a death as alcohol-related. The number of smoking-related deaths was estimated using the indirect method developed by Preston et al. (2010). The method is based on data from 21 high income countries for the period 1950–2007 and uses age- and sex-specific lung cancer death rates as indicators of the population level health damage from smoking. The method develops a regression model that uses lung cancer mortality to predict smoking-related mortality in other causes of death. The coefficients from this regression model and information on expected lung cancer death rates among non-smokers as well as the observed lung cancer mortality rates in the Swedish population are used to estimate the overall fraction of deaths attributable to smoking. The original method is restricted to ages 50 and above, ages at which most lung cancer deaths occur, and we rely on an extension of this method presented in Martikainen et al. (2014) to also cover ages between 30 and 49 years. For a more detailed account, see Preston et al. (2010). In our analyses, lung cancer death was defined as any death having lung cancer as the underlying or a contributing cause. Lung cancer deaths were identified as malignant neoplasms of either lung, trachea, or bronchus [162A, 162C-E, 162W, or 162X in ICD9 (1991–1996) and as C33, C34.0–3, or C34.8–9 in ICD 10 (1997–2008)].

The estimated number of alcohol- and smoking-related deaths and all-cause deaths were used to calculate mortality rates and life expectancies. Age standardized mortality rates (ASMR) were calculated to assess trends in alcohol and smoking-related mortality over the time period by education and sex. We also estimated temporary life expectancies (Chiang 1984; Preston et al. 2000) between the age 30 and 74. The temporary life expectancy reflects the average number of years lived within a set age bracket (Arriaga 1984; Chiang 1984), in this case 30–74.

To assess the contribution of alcohol and smoking to life expectancy, three sets of life expectancies were calculated by sex, period and education. First, observed life expectancy was calculated using the observed death rates. Second, life expectancy was re-calculated after excluding all alcohol-related deaths from the calculation of death rates. Third, life expectancy was re-calculated after excluding all smoking-related deaths. When calculating life expectancy without alcohol or smoking, deaths classified as alcohol and smoking-related, respectively, were right-censored. Thus, the time at risk remained the same in the different expectancy calculations, only the number of deaths differed.

## Results

Table 2 shows ASMR by sex, period and education for ages 30–74. The observed age distribution for the initial period (1991–1993) was used as the standard population. Smoking-related mortality declined among men in all educational categories while it increased among women. The fraction of mortality attributable to smoking among men remained largely stable through the study period, indicating that smoking-related mortality declined at a similar rate as all-cause mortality. The fraction of mortality attributable to smoking increased among women and in 2006–2008 smoking accounted for nearly 20% of all deaths. Smoking-related mortality was of similar magnitude among men and women in 2006–2008 while it was almost twice as high among men compared to women in 1991–1993. Among men, smoking-related mortality declined somewhat faster among those with low education, which lead the rate difference (RD) in smoking-related mortality to decrease during the study period. Among women, however, the increase in smoking-related mortality was faster among the low educated, leading to an increase in the RD between the high and low educated.

**Table 2:** Age-adjusted all-cause, smoking- and alcohol-related mortality per 100,000 person years by educational attainment, men and women, Sweden, 1991–2008

	Men						Women					
	All	AF^a^	High	Int.	Low	RD^b^	All	AF^a^	High	Int.	Low	RD^b^
Smoking-related mortality												
1991–1993	113	12.5	60	97	133	74	55	11.2	23	49	69	46
1994–1996	102	12.3	47	89	124	77	60	12.9	25	56	77	52
1997–1999	98	12.8	48	89	122	73	60	13.6	22	54	81	60
2000–2002	87	12.4	44	80	110	66	71	16.5	27	69	97	70
2003–2005	83	12.5	40	75	110	70	74	18.3	31	70	112	81
2006–2008	71	11.7	28	67	98	69	75	19.6	32	74	116	84
Change	−42	−0.8	−32	−30	−35	−5	+20	+8.4	+10	+25	+47	+38
Alcohol-related mortality												
1991–1993	53	5.9	17	47	72	54	10	2.1	6	10	14	9
1994–1996	51	6.1	18	47	69	50	11	2.3	6	11	15	9
1997–1999	48	6.2	18	48	66	48	11	2.4	6	11	16	10
2000–2002	48	6.8	19	49	67	47	12	2.7	7	12	18	11
2003–2005	51	7.8	22	54	71	49	13	3.1	6	13	22	16
2006–2008	49	8.2	23	53	69	46	13	3.3	7	14	20	14
Change	−4	+2.4	+6	+6	−3	−8	+3	+1.2	+1	+4	+6	+5

Age adjusted using the observed age structure in 1991–1993

^a^Attributable fraction (AF) in % of all-cause mortality

^b^Rate difference (RD) between the high and low educated

Alcohol-related mortality was higher among men compared to women throughout the study period. Among men, the alcohol-attributable fraction increased somewhat and a similar trend, at lower levels, was observed among women. Among women, alcohol-related mortality increased moderately among the low educated, while was stable among the high educated. Consequently, the RD in alcohol-related mortality increased. Among men, the trends were reversed, with a small increase among the high and intermediate educational groups and stable rates among the low educated, causing the rate difference to decrease.

**Fig. 1:**
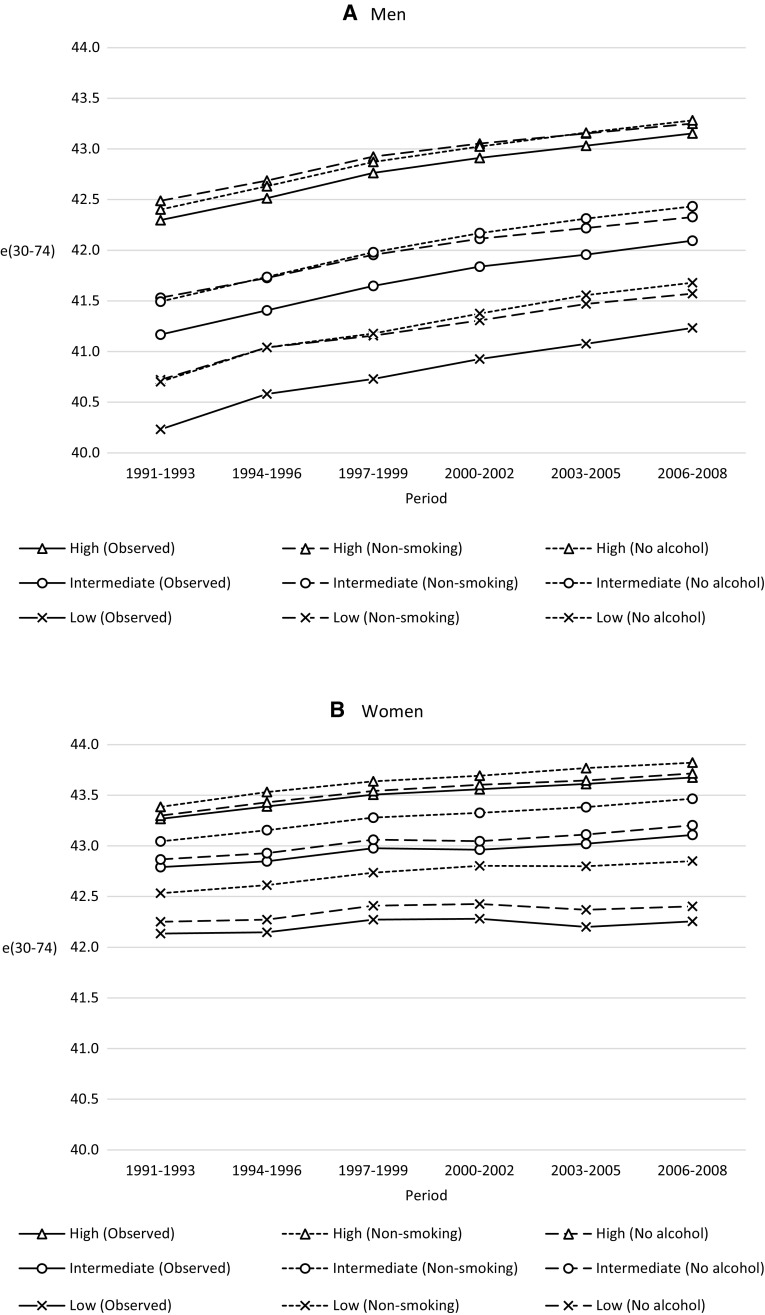
Temporary life expectancy (ages 30 and 74) for observed deaths, excluding smoking-related deaths, and excluding alcohol-related deaths by high, intermediate, and low educational attainment, Sweden, 1991–2008. Men and women

Figure 1a displays temporary life expectancies (30–74) for men by education. The solid lines represent the observed life expectancy while the dashed lines represent life expectancy when smoking-related deaths are excluded and the dotted lines represent mortality when alcohol-related deaths are excluded. The results show that educational inequalities in life expectancy can be partly attributed to smoking and alcohol. Alcohol and smoking contribute about equally to life expectancy while smoking makes a clearly larger contribution to mortality (Table 2). This is because alcohol-related deaths occur at younger ages [for an example see Finnish estimates (Martikainen et al. 2014)], thereby having a greater impact on life expectancy compared to ASMR. However, smoking and alcohol do not seem to contribute substantially to the time trends in life expectancy.

While both smoking and alcohol contribute to the educational inequalities in life expectancy among women (Fig. 1b), smoking clearly makes a larger contribution than alcohol. Observed life expectancy among low educated women only improved slightly during the study period. When excluding smoking-related deaths the development in life expectancy for low educated women is much closer to that of women with intermediate or high educational attainment.

**Fig. 2:**
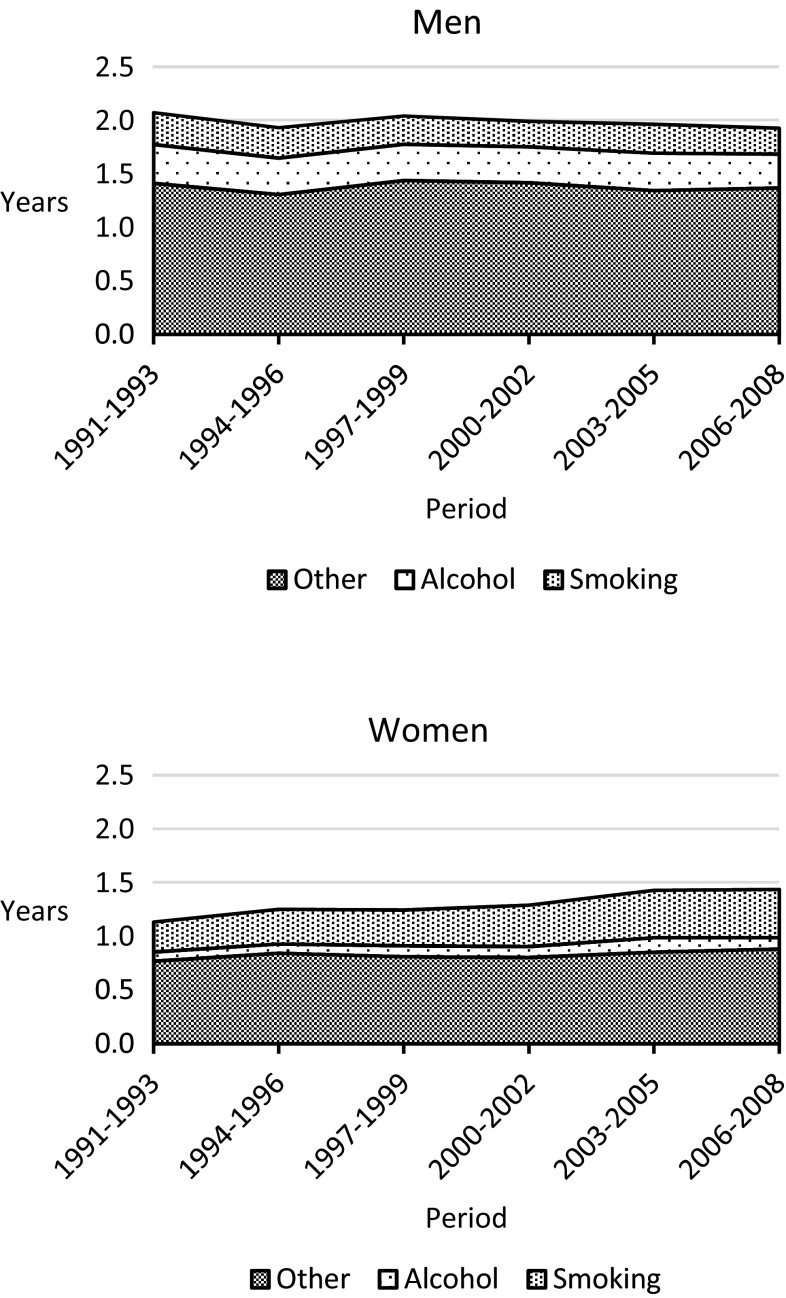
The contribution of smoking and alcohol-related mortality to the difference in temporary life expectancy (ages 30–74) between high and low educated, Sweden 1991–2008. Men and women

Figure 2 displays a decomposition of the educational gap in life expectancy between high and low educated. To avoid overlap, smoking-related deaths were estimated after removing alcohol-related deaths. While alcohol and smoking contribute to the educational gap in life expectancy, the major part of the gap is attributable to other causes of death. Among men, around 14% of the gap could be attributed to smoking-related mortality while around 17% could be attributed top alcohol-related mortality. These proportions remained stable over the study period. Among women, less than 8% of the gap could be attributed to alcohol-related mortality while the proportion attributable to smoking increased from 25 to 32% during the follow up. 58% of the increase in the educational gap in temporary life expectancy among women between 1991 and 2008 can be attributed to smoking.

## Discussion

### Summary of the main results

The results showed that from 1991 to 2008 both alcohol- and smoking-related mortality declined among men, but increased among women. The increases in alcohol- and smoking-related mortality among women were more pronounced among the low educated. The observed increase in smoking-related mortality among low educated women was the major cause for the modest gains in life expectancy in this group during the 1990s and 2000s and accounted for the majority of the increase in the educational gap in life expectancy. Alcohol-related mortality was higher among men compared to women. Among men alcohol and smoking contributed about equally to educational inequalities in mortality, but among women the contribution of smoking was clearly larger.

### Methodological considerations

When estimating the effect of harmful consumption of alcohol and smoking on mortality, there are advantages in using indirect methods based on cause of death, rather than relying on consumption data. The health effects of unhealthy behaviors are largely dependent on lifetime consumption habits, both in terms of quantity and patterns of consumption (Hiscock et al. 2012; Murray et al. 2002; Skov-Ettrup et al. 2011). However, consumption data from cross-sectional surveys may not accurately reflect life-time patterns and are subject to self-report biases and non-response. A benefit of using cause of death data is that it reflects lifetime exposure. A drawback is that it is unclear whether results are driven by levels or patterns of consumption.

The contribution of alcohol- and smoking-related mortality varies greatly with age (Martikainen et al. 2014). Therefore, it is difficult to directly compare estimates from sources using different age ranges. However, where comparable, the estimates on lung cancer mortality and the contribution of smoking- and alcohol consumption to total mortality yielded in this study were similar to other studies (Agardh et al. 2015; Preston et al. 2010; The National Board of Health and Welfare 2014). The majority of smoking- and alcohol-related deaths occur within the age-span of this study (30–74 years) (Foulds et al. 2003; Martikainen et al. 2014; The National Board of Health and Welfare 2014), which implies that using other age spans would result in smaller proportions of alcohol- and smoking-related deaths.

The cause of death register has near complete coverage (less than 1% missing). For each death, a medical investigation is conducted and the cause of death is reported by a physician in the death record (The National Board of Health and Welfare 2010). The accuracy of the cause of death is then dependent on the accuracy of investigations carried out by individual physicians. Validation studies have shown that while there are some uncertainties in the accuracy for individual cause of death codes (particularly among the oldest-old), the cause of death register provides accurate data at the population level for the calculation cause-specific death rates (Johansson and Westerling 2000; The National Board of Health and Welfare 2010).

### Interpretation and significance of results

The results demonstrate that behavioral factors partly explain the persistence and trends of social inequalities in mortality in welfare states. Alcohol and smoking contributed to educational inequalities in mortality both among men and women, and smoking made a major contribution to the increase in educational inequalities in mortality among women. However, the majority of the educational gap in life expectancy could not be explained by alcohol and smoking, indicating that other factors is important as well. In contrast to results from similar studies in Denmark (Koch et al. 2015) and Finland (Martikainen et al. 2013), where levels and time trends in social inequalities in life expectancy were shown to be mainly attributable to alcohol and smoking (Koch et al. 2015; Martikainen et al. 2013), alcohol- and smoking-related mortality were substantially less important for time trends in Sweden. The results show that even though patterns in educational inequalities in mortality are relatively similar across the Nordic countries (Mackenbach et al. 2016; Shkolnikov et al. 2012), the specific mechanisms generating the patterns differ between the countries, especially among women. This finding calls for direct comparative research within the Nordic countries, but also elsewhere.

The use of wet smokeless tobacco (snus) is more common among Swedish men than among women (Furberg et al. 2006) and has been suggested as part of the explanation for the relatively low smoking rates (Foulds et al. 2003) among men. Younger cohorts have been found to be more likely to use snus among both men and women (Furberg et al. 2006). One Swedish study (Furberg et al. 2006) found that low educated men were more likely to use snus while no educational gradient was found among women (Furberg et al. 2006). It is possible that the long-established and widespread consumption of snus, used as a partial substitute for cigarette smoking, has attenuated the educational gradient in smoking-related mortality over time, particularly among men. However, both genders are likely to benefit from this substitution as switching from smoking to snus is a switch from a more to a less health damaging product. The Preston et al. (2010) approach to estimate smoking-related mortality is based on the incidence of lung cancer mortality, and it has been a repeated finding that the use of snus is not a risk factor for lung cancer (Boffetta et al. 2008). The results presented in this study should then be interpreted as estimates of smoking-related mortality and do not cover any potential damage from other forms of tobacco use.

While men are consistently reporting higher levels of binge drinking in surveys than women, results display only modest educational differences in problem drinking (Bloomfield et al. 2006; Backhans et al. 2015). Setting aside the real possibility that problem drinking may be difficult to capture in surveys and assuming that problem drinking is in fact relatively evenly distributed across educational groups, the fact that alcohol-related mortality was found to contribute to educational inequalities in mortality present a contrast to this finding. One explanation for this discrepancy is that while mortality risk is an outcome of the total effect of alcohol across the life-course, cross-sectional survey data may instead reflect a single dimension of alcohol use at one point in time. Furthermore, the low educated have been found to be more likely to engage in multiple negative health behaviors (Laaksonen et al. 2003) and have worse health in general (Mirowsky and Ross 2003). The low educated may then experience more severe health consequences from the same level of drinking or smoking. Differences in mortality attributable to alcohol (and smoking) are likely to reflect differences in behavior across educational groups, but also differences in susceptibility.

### Conclusions

Alcohol and smoking-related mortality contributed significantly to educational inequalities in life expectancy among Swedish men and women in the 1990s and 2000s. Alcohol and smoking contribute equally (about 15%) to inequalities among men, while alcohol only makes a modest but increasing contribution to inequalities among women. Smoking-related mortality declined among men in all educational groups while it increased among women. Smoking-related mortality accounted for the majority of the increase in educational inequalities in life expectancy among women. The modest gains in life expectancy observed among low educated women during recent decades may to a substantial degree be attributed to the harmful effects of smoking.
